# New Media Literacy, Health Status, Anxiety, and Preventative Behaviors Related to COVID-19: A Cross-Sectional Study in Taiwan

**DOI:** 10.3390/ijerph182111247

**Published:** 2021-10-26

**Authors:** Shih-Chieh Hung, Shu-Ching Yang, Yi-Fang Luo

**Affiliations:** 1 The Intelligent Electronic Commerce Research Center, Institute of Education, National Sun Yat-Sen University, Kaohsiung 80424, Taiwan; kiwi2234@hotmail.com; 2 Department of Information and Communication, Southern Taiwan University of Science and Technology, Tainan 710301, Taiwan; 3 Center for Teaching and Learning Development, National Kaohsiung University of Science and Technology, Kaohsiung 805301, Taiwan

**Keywords:** COVID-19, new media health literacy, health, anxiety, preventative behaviors

## Abstract

Internet media may exacerbate public confusion and anxiety about COVID-19. New media health literacy (NMHL) is considered to play a protective role against health-related misinformation from the media for individuals to maintain their health. The current study aims to examine the relationship among Taiwanese adults’ NMHL, health status, anxiety, and prevention behaviors during the COVID-19 pandemic. This cross-sectional study was conducted through an online survey, and 342 responses were included in the analysis. The survey tools include Health Status, COVID-19-Related New Media Health Literacy, COVID-19 Anxiety, and COVID-19 Preventive Behaviors. The research showed that both functional and critical prosuming literacy had positive relationships with health status. Functional consumption literacy had a weak negative correlation with COVID-19 anxiety. Furthermore, critical consumption literacy had a positive relationship with COVID-19 preventive behaviors. Therefore, individuals’ health, anxiety, and prevention behaviors are affected by different aspects of COVID-19-related new media health literacy. Compared to their consuming media literacy, Taiwanese adults have insufficient prosuming media literacy in regard to COVID-19 health issues.

## 1. Introduction

In December 2019, novel coronavirus disease 2019 (COVID-19) was discovered and started to spread worldwide. On January 30, 2020, the World Health Organization (WHO) further defined the COVID-19 pandemic as a Public Health Emergency of International Concern (PHEIC), which caused a large amount of public concern and fear about the possibility of a pandemic [1]. The popularity of the internet prompted users to seek information in real time and maintain a high level of attention to COVID-19-related information. According to several studies, due to this physical threat and the social and physical distancing context, the most common source for the acquisition and exchange of COVID-19 health information was internet media, which grew at an unprecedented scale [2,3,4,5]. 

### 1.1. Social Media Use and Health

Internet media can provide fast and critical guidance on important public health messages by raising awareness and promoting positive health behaviors, which can help reduce disease transmission [6]. However, internet media, especially social media, may be a source of conflicting and rapidly disseminated misinformation related to COVID-19, exacerbating public confusion and affecting individuals’ mental states [4,7,8,9]. Gao et al. (2020) investigated the social media use and mental health problems of 4,872 participants from 31 provinces and autonomous regions in China during the COVID-19 pandemic and found that 82.0% of participants had frequent exposure to social media, which was associated with a higher level of anxiety [10]. Individuals attempt to clarify their suspicions about COVID-19 through social media; however, this can lead to an overload of (mis) information, thereby increasing anxiety and depression that may influence mental health [4,7,11]. 

### 1.2. Media Health Literacy

Media health literacy, combining the concepts of media literacy and health literacy, is considered to play a protective role, protecting individuals from health-related misinformation from the media [12,13]. Although media health literacy is an emerging concept with a lack of empirical data support [12], it is still possible to understand its importance from the perspectives of media literacy or health literacy. 

Media literacy is the ability to access, analyze, assess, create, and share media and to develop critical thinking to reduce vulnerability to negative media influences and enhance health outcomes [14,15]. The connotation of media literacy varies with the form of media, from the reading and writing capabilities corresponding to traditional media to the new media literacy corresponding to online social media [16]. Unlike traditional media users who engage in passive consumption, new media users emphasize the active construction, creation, and sharing of media content [15].

Chen et al. (2011) proposed a promising framework of new media literacy that contains four components: functional consuming (FC) literacy, critical consuming (CC) literacy, functional prosuming (FP) literacy, and critical prosuming (CP) literacy [17]. FC literacy refers to the ability to access new media and understand the literal meaning of media content. CC literacy involves the ability to deconstruct media messages, remix media content, and reconstruct media messages by integrating one’s own views and to question, criticize, and challenge the credibility of media content. FP literacy involves the ability to produce/create media content, disseminate information at hand, and process media content across multiple modalities. CP literacy focuses on the ability to participate interactively and critically in new media environments and create media content with a critical understanding of embedded sociocultural values and ideological issues [18,19].

Media literacy is closely related to health literacy when the media become resources for health information [20]. The common definition of health literacy is individuals’ cognitive and social abilities to acquire and comprehend health information and make wise and proper health-related decisions on this basis [21]. Nutbeam (2008) proposed the structural concept of health literacy, including functional, interactive, and critical levels [22]. Functional health literacy describes basic-level skills of reading and writing for health information as well as the possession of basic hygiene and health knowledge. Interactive health literacy refers to personal communicative and social skills that are applied to extract health information and learn significance from different forms of health communication. Critical health literacy involves the most advanced cognitive abilities of an individual, which can be applied to critically analyze information and rely on high-quality information to make intelligent decisions about health.

The conceptual assumption of health literacy is different from that of media literacy. Health information is usually purposely produced by the health system, while mass media content is usually implicit and may promote or damage health. Therefore, the single concept of either health literacy or media literacy seems insufficient to explain how individuals master health information in the media context [13]. Building on the synthesis of health literacy and media literacy, Levin-Zamir et al. (2011) further proposed the concept of media health literacy and conceptualized it as a continuum, ranging from (1) the ability to comprehend health-related content in the various forms of media (equivalent to functional health literacy); (2) critically scrutinize health information from a wide range of media sources and information (equivalent to critical health literacy), and (3) express intention to respond with health-related actions to more interactive, accessible, and structured communication media, including personal behavior or public action (equivalent to interactive health literacy) [13,20]. These concepts of functional, critical, and interactive media health literacy can be aligned with FC literacy, CC literacy, and prosuming literacy (including functional and critical prosuming literacy), respectively, proposed by Chen et al. (2011) [17].

### 1.3. Media Health Literacy and Health

Although few empirical studies have explored health and media literacy, media literacy is regarded as playing an important role in how individuals obtain health information through various forms of media and how they understand and use this information [12]. Media literacy in the context of health, that is, media health literacy, can help individuals identify, analyze, communicate, and participate in health-related information and discourse through multimodal media and is even empowered to adopt, advocate, or maintain health-related actions/reactions [12]. In other words, media health literacy has the potential to improve the translation of changes in beliefs into changes in behavior. According to the message interpretation process (MIP) model, individuals internalize messages via the combination of logic and emotional processes, thereby influencing behavior [23] and subsequent changes from behavior. The internalization of media health messages involves the concept of media health literacy. Levin-Zamir et al. (2011) showed that media health literacy has a positive relationship with health outcomes and behavior [13].

According to the survey [24], the proportion of Taiwanese using social media exceeds 80%. However, given that frequent new media (especially social media) exposure is closely related to a higher prevalence of mental health problems (e.g., [4,7,8,9,10]), the impact of new media health literacy (NMHL) on individuals’ mental health outcomes through the COVID-19 pandemic has not yet been fully examined. Therefore, exploring Taiwanese new media health literacy related to COVID-19 and health issues is the reason for this study.

The survey method of self-report scale has been adopted by many researchers (e.g., [15,18,21,25,26]) to understand individuals’ health literacy and media literacy. The belief in adopting the self-report scale is that the information provided by respondents’ self-reports is often more accurate than the indirect information regarding the respondents provided by others, because respondents themselves are much closer to the issues in question than others. However, self-reporting reports may have some limitations, such as social desirability bias, (non-) acquiescent bias, respondents’ different interpretations of questions, and fixed options that restrict sufficient and flexible expression [27]. Nevertheless, when social distancing becomes a policy norm during the COVID-19 pandemic, the implementation of self-reporting scales through the Internet is a feasible research method.

Therefore, this study aims to design and validate the COVID-19-related New Media Health Literacy Scale based on the above-mentioned media (health) literacy framework [13,17], and then, it examines Taiwanese people’s new media health literacy related to COVID-19 and its relationship with health-related variables, including health status, COVID-19 anxiety, and COVID-19 preventive behaviors.

## 2. Materials and Methods

### 2.1. Participants and Procedures

This cross-sectional study aims to develop the COVID-19-related New Media Health Literacy Scale and explore its relationship with health-related variables. According to the recommendations of Wu and Tu (2011) and Comrey (1973), the appropriate sample size for scale factor analysis is more than 300 [28,29].

Regarding the implementation procedures, as the Taiwanese government has recommended specific preventive measures during the pandemic, including precautions regarding close contact and touching, we designed an anonymous online questionnaire in the traditional Chinese language to invite potential respondents. Respondents completed the questionnaire through an online survey platform (Google Forms). We published the URL of the online questionnaire on social networks (Plurk and Facebook) and explained the purpose of the research in order to recruit interested users to fill out the online questionnaire. In addition, we invite users to forward information freely. 

On the front page of the questionnaire, we provided informed consent information, so that the respondents started to fill out the questionnaire only after agreeing to participate in the research. Gift certificates or the chance to win money was offered for their participation. The inclusion criteria of the participants included (i) having access to a smartphone and social networking sites, and (iv) a willingness to take part in the survey. The participants who did not entirely complete the survey were excluded from the final sample. The data collection was conducted over three days (from 25 to 27 April 2020). Finally, a total of 342 participants responded to the questionnaire (at the 95% confidence level, the confidence interval is 5.3). 

### 2.2. Instrument

#### 2.2.1. Health Status

Health status was measured by asking the participants how they felt about their overall health using a 5-point Likert scale with the options very good (5), good (4), moderate (3), poor (2), and very poor (1). This measure is a widely used, validated indicator of health in the field of social sciences [30]. In this study, most of the participants rated their level of health as 4 (40.35%) or 5 (39.18%). In other words, the participants’ self-rated health tended to be good (*M* = 4.17, *SD*= 0.79).

#### 2.2.2. COVID-19-Related New Media Health Literacy (COVID-19 NMHL) Scale

The COVID-19 NMHL Scale was designed to measure the participants’ critical and active access to and their analysis, evaluation, construction, creation, and sharing of new media information related to COVID-19. This scale was developed based on the new media literacy framework proposed by Chen et al. (2011) [17] and a thorough review of the literature [13,17,18,19,22]. The items of the COVID-19 NMHL Scale were answered on a 5-point Likert scale ranging from 1 (strongly disagree) to 5 (strongly agree). We were interested in identifying the specific subcomponents constituting the concept of COVID-19-related new media health literacy. Specifically, we aimed to determine whether, as predicted, the scale could be broken down into components that were similar to those identified by Chen et al. (2011) [17]; therefore, we elaborate on the components of the COVID-19-related NMHL Scale in the results section.

#### 2.2.3. COVID-19 Anxiety

The COVID-19 anxiety measure was used to examine the participants’ complex emotional responses to potential COVID-19 infection, such as tension, fear, panic, and anxiety [5]; the scale included four items: (1) I think I have a high probability of being infected with COVID-19; (2) I feel that I am very likely to be exposed to individuals with suspected or possible cases of COVID-19; (3) I think that if I go out, I will likely be infected with COVID-19; and (4) I feel that even if protective measures are taken, I may still be infected with COVID-19. The items were answered using a 5-point Likert scale with scores ranging from 1 (strongly disagree) to 5 (strongly agree). Higher scores represented a higher level of anxiety and worry about the COVID-19 pandemic. Exploratory factor analysis (EFA) showed that the Kaiser–Meyer–Olkin (KMO) test value was 0.72, and that the Bartlett test for sphericity was significant (χ^2^ = 521.84, *p* < 0.001). The outcome of the EFA indicated a single-factor structure (α = 0.81), with factor loadings ranging from 0.74 to 0.84, and the explained variance was 64.25%.

#### 2.2.4. COVID-19 Preventive Behaviors

The items used to measure COVID-19 preventive behaviors assessed the participants’ degree of personal health actions to prevent COVID-19 infection; the four measurement items were developed based on the relevant literature (e.g., [1,5]): (1) I avoid gatherings; (2) I socially distance from others; (3) I maintain the hygienic habit of washing my hands frequently; and (4) I wear a mask in indoor public places. The items were answered on a 5-point Likert scale ranging from 1 (strongly disagree) to 5 (strongly agree). The EFA showed that the KMO test value was 0.75 and that the Bartlett test for sphericity was significant (χ^2^ = 538.13, *p* < 0.001). The outcome of the EFA indicated a single-factor structure (α = 0.82), with factor loadings ranging from 0.52 to 0.72, and the explained variance was 65.93%.

### 2.3. Ethical Issues

This study followed the code of research ethics and conformed to the Taiwan government’s institutional review board rules for exempt review. We did not collect any relevant identifying information of the humans involved, and an anonymous design questionnaire was used in this study. The questionnaire instructions clearly informed the participants of the research purpose and their rights regarding joining or dropping out of this study. Four checkpoints are provided in the research design so that respondents can stop answering at any time: (1) From the invitation and introduction SMS/email link to the online questionnaire; (2) After reading the research purpose, respondents decided to fill in the questionnaire; (3) During online filling-in, respondents are noted to quit at any time if they would like to; (4) Upon completing the entire questionnaire, and finally confirm “Send”. Respondents were assured that their participation was voluntary, anonymous, and strictly confidential and that they had the right to refuse to participate in the study at any time without any penalty.

### 2.4. Data Analysis

All analyses were performed using Statistical Product and Service Solutions 22.0 (SPSS) (IBM Corp, Armonk, NY, USA) software. First, the KMO measure of sampling adequacy and Bartlett’s test of sphericity were used to assess the suitability of the data for factor analysis [31]. We adopted the principal component analysis method with varimax rotation and eigenvalues >1 for EFA. Second, descriptive statistics, including the mean and standard deviation, were used to describe the new media health literacy data. Third, repeated-measures ANOVA and Pearson correlation analysis were performed to analyze the four aspects of new media health literacy. Finally, multiple regression analysis was conducted to explore the relationships among new media health literacy and health status, anxiety, and preventive behaviors.

## 3. Results and Discussion

### 3.1. General Profile of Participants

An overview of the frequencies of the demographic variables of the final sample (n = 342). A total of 342 participants with a mean age of 23.42 (*SD* = 7.94) were included in the final analysis, and their ages ranged from 18 years old to 70 years old. Among the respondents, 146 (42.69%) were male, and 196 (57.31%) were female. Regarding their education level, 6.14% had a high school education, 72.81% had a university education, 20.17% had a master’s education, and 0.88% had a doctoral education.

### 3.2. COVID-19-Related New Media Health Literacy

The COVID-19 NMHL Scale developed in this study is based on the refined frame-work of new media literacy proposed by Chen et al. (2011) [16]. To define the specific subcomponents constituting the concept of COVID-19-related NMHL, we performed an EFA and then assessed the reliability of each separate subscale that emerged from the factor analysis. The value of the KMO test of sampling adequacy was 0.91, and Bartlett’s test of sphericity was significant at 6104.24 (*p* < 0.001), indicating that the items were appropriate for factor analysis. Therefore, we performed a principal component factor analysis using the varimax rotation method with Kaiser normalization. Table 1 shows that the factor analysis performed in SPSS yielded four factors with eigenvalues above 1.00. The factor loadings ranged from 0.70 to 0.93, and the total explained variance was 74.27%. In descending order by variance, the subcomponents that emerged were critical prosuming health literacy (CP, α = 0.95), critical consuming health literacy (CC, α = 0.92), functional consuming health literacy (FC, α = 0.90), and functional prosuming health literacy (FP, α = 0.87).

FC health literacy referred to the ability to understand COVID-19-related content in the media and contained five items. CC health literacy referred to the ability to critically analyze COVID-19-related content and contained seven items. FP health literacy contained three items related to the ability to disseminate and produce COVID-19-related media content and process COVID-19-related media content across multiple modalities. CP health literacy contained seven items focusing on the ability to interact and participate critically in the context of new media and critically analyze COVID-19-related media content.

The descriptive statistics of the COVID-19 NMHL Scale are also presented in Table 1. Table 1 shows that FC literacy had the highest mean score (*M* = 4.37, *SD* = 0.58). Among the FC literacy items, the participants reported the highest level of agreement that they knew how to find information about COVID-19 pm online media. The lowest score for the four aspects of new media literacy was found for CP literacy (*M* = 1.90, *SD* = 0.89). Among the CP literacy items, the participants had the lowest level of agreement that they could edit COVID-19 content presented as digital information and put it on the internet.

In addition, the repeated-measures ANOVA showed significant differences among the scores of the four aspects of new media literacy (*F =* 885.92, *p* < 0.001, $\eta_{p}^{2}=0.72$). The post hoc tests revealed that the CP literacy score (*M* = 1.90, *SD* = 0.89) was significantly lower than the scores of the other three aspects of COVID-19 NMHL. The FP literacy score (*M* = 3.38, *SD* =0.90) was significantly lower than the FC (*M* = 4.37, *SD* = 0.58) and CC (*M* = 4.02, *SD* = 0.65) literacy scores. The FC literacy score was significantly higher than the scores of the other three aspects of COVID-19 new media health literacy.

The participants in this study had the highest FC literacy, which was followed by CC literacy and then FP and CP literacy. To some extent, this finding supports the claim of Chen et al. (2011) that the development of personal media literacy occurs along two continuums, one from consumer literacy to production literacy and one from functional literacy to critical literacy [16]. Consuming literacy is the capability to read media content, and in addition, functional literacy focuses on the capability of knowing relevant skills and knowledge, and critical literacy focuses on the capability of meaning making and judging the credibility and usefulness of information [32]. Functional literacy is the foundation of critical literacy, and consuming literacy precedes prosuming literacy [19].

Virtue’s study (2020) showed a similar result; that is, when taking a web literacy test, college students self-reported that they were best at reading-related skills, especially basic reading skills [33]. However, they self-reported being the least skilled in writing-related skills. Although the inconsistency of students’ reading and writing development may lead to differences in consuming literacy and prosuming literacy [34], a lack of training in writing skills may also lead to individuals’ prosuming literacy being not as good as their consuming media literacy. Kim and Kim (2021) pointed out that the current common educational methods that focus on students becoming receivers of knowledge rather than active contributors and producers may cause students to lack training in writing skills, which may make it difficult for them to express their thoughts and ideas through the media [35].

Finally, the Pearson correlation analysis results are presented in Table 2. Table 2 shows that FC literacy and CC literacy had a strong significant correlation (*r* = 0.57, *p* < 0.001). CC literacy and FP literacy had a moderately significant correlation (*r* = 0.35, *p* < 0.001). FC literacy and FP literacy had a small significant correlation (*r* = 0.25, *p* < 0.001). FP literacy also had a small significant correlation with CP literacy *(r* = 0.29, *p* < 0.001) (Cohen, 1988) [36]. However, there were nonsignificant correlations between CP literacy and FC (*r* = −0.09, *p* = 0.10) and CC (*r* = 0.08, *p* = 0.16) literacy.

These findings indicate that the correlations between reading and writing are far from perfect. Reading and writing consist of both dependent and independent abilities, and independent abilities allow reading and writing not only to be learned separately but also to have large numbers of variables and a limited amount of shared variance [34,37]. Prosuming literacy related to writing skills involves sharing one’s own opinions with others [38]. Previous research has found that factors that affect internet users’ sharing behavior include self-interest, communal incentives, type of content [39], status seeking, entertainment, and habitual diversion [40]. In particular, critical literacy is a reflection of the individual’s capacity to contextualize knowledge, in particular with an independent character [41]. Therefore, although some scholars believe that consuming literacy is integrated with prosuming literacy given that individuals need to develop consuming literacy before developing prosuming literacy-related abilities [17,18,19], prosuming literacy may also be affected by other factors. This reduces the relationship between prosuming literacy (especially CP literacy) and consuming literacy.

### 3.3. Relationships between COVID-19 New Media Health Literacy and Health

The results of the multiple regression analysis are presented in Table 3. Table 3 shows that health status was significantly positively related to FP literacy (Beta = 0.16, *p* = 0.005) and CP literacy (beta = 0.11, *p* = 0.04). FC literacy was significantly negatively related to anxiety (Beta = −0.14, *p* = 0.04). CC literacy was significantly positively related to COVID-19 preventive behaviors (Beta = 0.28, *p* < 0.001). These findings suggest that individuals’ development into functional and critical prosumers who interactively and critically disseminate and produce COVID-19-related media content serves to maintain their good health status. Existing research indicates that prosumption capacity is the ability to integrate production and consumption and that prosumers can cultivate their ability to access, understand, and process health information while being able to manage their own health conditions [42].

In addition, in this study, FC literacy had a weak negative correlation with anxiety. FC literacy is an ability to access media content and understand its textual meaning [16]. Individuals with low FC health literacy are less able to effectively comprehend health information and seek proper clarifications for health problems, which in turn increases their anxiety about health problems [43]. However, information accessibility can help individuals reduce their uncertainty and anxiety in the case of new health issues or stressful situations [44]. Recent research also found that the ability to access and understand information can significantly reduce coronavirus anxiety [26]. COVID-19, as an emerging large-scale infectious disease, has caused many unpredictable situations worldwide. When an event or situation is interpreted as unpredictable and uncontrollable, it will cause the individual to generate anxiety [45]. Improving the individual’s understanding of the content of the event can reduce the anxiety caused by the unknown [46].

There was a significant positive correlation between CC literacy and COVID-19 preventive behaviors, and CC literacy predicted COVID-19 preventive behaviors by approximately 12%. This finding suggests that CC literacy (understanding COVID-19 and accordingly making judicious decisions) is the key to success in adopting COVID-19 preventive behaviors. People who are considered to have high critical health literacy can analyze information critically to help use high-quality information and make informed decisions about health to better control life events and situations [22]. Aller, Fauth, and Seedall (2021) found that the intervention of health literacy education courses on how to identify high-quality, evidence-based resources can improve college students’ sense of self-efficacy in adopting preventive health behaviors [47].

## 4. Limitations

This study had some limitations. As a result of individual subjectivity, the participants’ self-reports may not reflect their actual media health literacy. Furthermore, since the online survey in this study was distributed through social networks, this may exclude non-social network users, resulting in under-representation of the sample and coverage errors. The online questionnaire cannot assess the number and characteristics of rejected respondents, and may also cause non-response errors, which leads to the limitations of sample selection in this study.

In addition, although in a statistical sense, media health literacy can predict health, including health status, anxiety, and health behavior, in a practical sense, these variables are related but not necessarily causally related. Therefore, other research methods, such as experiments or observations involving longitudinal data collection and analyses of variables, can be used in future research to further examine media health literacy and other related health variables. Another limitation of this study was that it did not investigate the participants’ experience with COVID-19, such as whether there had been suspected or confirmed cases in their residential communities, which may interfere with anxiety and healthy behaviors.

Finally, it should be noted that the survey period of this study was conducted before Taiwan raised its pandemic alert to the third level, when the impact of the COVID-19 pandemic was not serious compared to other countries. Therefore, temporal and spatial background factors might mitigate or moderate the investigation of emotional attitudes and the measurement of COVID-19 MHL, which should be taken into interpretation of our findings.

## 5. Conclusions

Creating a link between the framework of new media literacy and health literacy and the further development of the COVID-19 NMHL Scale to measure participants’ four types of related new media health literacy are the unique contributions of this study, which provide a crucial starting point and insight into this research field.

The current study revealed that compared to their consuming media literacy, Taiwanese adults have insufficient prosuming media literacy in regard to COVID-19 health issues. The findings of this research have practical implications for health education practice. The study showed that media literacy advocacy should focus more on how to produce and criticize media content to support the ability of the public to actively and critically participate in new media platforms. That is, the education sector and the government should cultivate individuals’ ability to jointly explore and reflect on problems through collective participation, to detect various meanings of different COVID information, to show concern for and engagement in public issues through dialogue, and to achieve the goal of promoting their health.

However, the study does not suggest that consumption literacy related to COVID-19 information is not important. Individuals’ health, anxiety, and prevention behaviors are affected by different aspects of COVID-19 new media health literacy. Therefore, implementing instructional programs that can enhance individuals’ different levels of health literacy may help reduce the health crisis caused by COVID-19, especially anxiety; maintain individuals’ good health status; and encourage individuals to adopt COVID-19 preventive behaviors.

In this way, future programs can intervene and make individuals consumers and prosumers, nurturing the four levels of health literacy and encouraging them to be more committed to participating more actively and critically in the consumption and production processes of digital media. In addition, future studies can further corroborate the effectiveness of interventions to transform audiences from pure media receivers to producers with media citizenship.

## Figures and Tables

**Table 1:** The factor loadings and descriptive statistics of the COVID-19 NMHL Scale.

Item	Factor Loadings				*Mean*	*SD*
	F1	F2	F3	F4		
**CP health literacy**					1.90	0.89
1. I gather and summarize the pros and cons of COVID-19 information and provide it to netizens.	0.93	0.01	−0.05	0.03	1.81	0.98
2. I summarize COVID-19 information and produce it into an online informative summary or FAQ to let netizens know more about COVID-19.	0.93	0.00	−0.08	0.05	1.83	1.01
3. I edit COVID-19 content presented as digital information and put it on the Internet.	0.92	0.04	−0.10	0.08	1.75	0.91
4. I call on netizens to discuss and criticize related issues caused by COVID-19 on the Internet.	0.90	0.00	−0.04	0.05	1.81	0.99
5. I actively participate in online discussion forums or platforms related to COVID-19 and express opinions.	0.84	0.00	0.05	0.20	1.99	1.03
6. I responded to netizens’ views on COVID-19 and propose my own views for their reference.	0.83	0.12	−0.03	0.13	2.07	1.05
7. Regarding inappropriate reports of COVID-19, I take the initiative to complain in the media to reflect opinions or request corrections.	0.81	0.07	−0.05	0.09	2.06	1.03
**CC health literacy**					4.02	0.65
1. I compare the COVID-19 information published by different media organizations.	0.06	0.84	0.15	0.08	3.88	0.90
2. I try to think about the COVID-19 information released by the media from a different perspective.	0.06	0.83	0.15	0.07	3.97	0.83
3. I try to compare information from different sources to get a clearer grasp of COVID-19 related information.	0.06	0.81	0.22	0.08	4.02	0.90
4. I synthesize old and new information to better understand COVID-19.	0.05	0.77	0.23	0.14	3.94	0.81
5. When I look at COVID-19-related information, I think about whether it has a specific position or point of view.	−0.01	0.76	0.23	0.06	4.16	0.80
6. When I look at the information related to COVID-19, I think about whether it has a one-sided bias.	−0.07	0.73	0.32	0.09	4.17	0.73
7. I analyze the positive and negative effects of COVID-19 information released by the media on individuals.	0.10	0.70	0.24	0.22	3.94	0.75
**FC health literacy**					4.37	0.58
1. It is easy for me to use various media to get information about COVID-19.	−0.12	0.19	0.86	0.11	4.49	0.64
2. I know how to find information about COVID-19 in online media.	−0.09	0.29	0.81	0.09	4.53	0.60
3. I can search for the COVID-19 information I want to know on the most suitable information platform.	0.03	0.30	0.81	0.06	4.24	0.76
4. I find accurate answers to my questions about COVID-19 through online media.	0.04	0.30	0.76	0.10	4.17	0.75
5. It is not difficult for me to grasp dynamically updated information about COVID-19.	−0.16	0.28	0.75	0.04	4.40	0.67
**FP health literacy**					3.38	0.90
1. I have the ability to integrate COVID-19 information across media (e.g., text, video or audio).	0.11	0.16	0.08	0.90	3.43	1.00
2. I have the ability to text or images to produce COVID-19 media content.	0.10	0.16	0.13	0.88	3.65	1.04
3. Regarding COVID-19 information, I can use proper media expressions to attract public attention.	0.31	0.18	0.12	0.77	3.04	0.97

**Table 2:** Correlation matrix of the four aspects of COVID-19 new media health literacy.

	FC	CC	FP	*M*	*SD*	*RANK*
FC	-			4.37	0.58	1
CC	0.57 ***	-		4.02	0.65	2
FP	0.25 ***	0.35 ***	-	3.38	0.90	3
CP	−0.09	0.08	0.29 ***	1.90	0.89	4

*** *p* < 0.001.

**Table 3:** Multiple regression analysis of COVID-19 new media health literacy and health.

	Health	Health Status		COVID-19 Anxiety		COVID-19 Preventive Behaviors	
NMHL		*Beta*	*t*	*Beta*	*t*	*Beta*	*t*
FC		0.06	0.97	−0.14	−2.08 *	0.07	1.06
CC		0.10	1.57	0.03	0.45	0.28	4.30 ***
FP		0.16	2.79 **	<0.001	0.02	0.06	0.97
CP		0.11	2.06 *	0.10	1.79	−0.05	−1.00
		*R* = 0.30 *R*^2^ = 0.09 *F* = 8.25 ***		*R*= 0.17 *R*^2^ = 0.03 *F* = 2.49 *		*R* = 0.34 *R*^2^ = 0.12 *F* = 11.34 ***	

* *p* < 0.05. ** *p* < 0.01. *** *p* < 0.001; *Beta*: standardized coefficients.
